# Construction of Metabolism Prediction Models for CYP450 3A4, 2D6, and 2C9 Based on Microsomal Metabolic Reaction System

**DOI:** 10.3390/ijms17101686

**Published:** 2016-10-09

**Authors:** Shuai-Bing He, Man-Man Li, Bai-Xia Zhang, Xiao-Tong Ye, Ran-Feng Du, Yun Wang, Yan-Jiang Qiao

**Affiliations:** 1 Key Laboratory of Traditional Chinese Medicine-Information Engineer of State Administration of Traditional Chinese Medicine, School of Chinese Pharmacy, Beijing University of Chinese Medicine, Beijing 100102, China; wenyuxuan2530@163.com (S.-B.H.); limanman@bucm.edu.cn (M.-M.L.); yexiaotongs@163.com (X.-T.Y.); duranfeng@126.com (R.-F.D.); 2 College of Chinese Medicine, Hebei University, Baoding 071002, China; baixia575@163.com

**Keywords:** CYP3A4, CYP2D6, CYP2C9, microsomal metabolic reaction system, metabolism prediction, classification, feature selection

## Abstract

During the past decades, there have been continuous attempts in the prediction of metabolism mediated by cytochrome P450s (CYP450s) 3A4, 2D6, and 2C9. However, it has indeed remained a huge challenge to accurately predict the metabolism of xenobiotics mediated by these enzymes. To address this issue, microsomal metabolic reaction system (MMRS)—a novel concept, which integrates information about site of metabolism (SOM) and enzyme—was introduced. By incorporating the use of multiple feature selection (FS) techniques (ChiSquared (CHI), InfoGain (IG), GainRatio (GR), Relief) and hybrid classification procedures (Kstar, Bayes (BN), K-nearest neighbours (IBK), C4.5 decision tree (J48), RandomForest (RF), Support vector machines (SVM), AdaBoostM1, Bagging), metabolism prediction models were established based on metabolism data released by Sheridan et al. Four major biotransformations, including aliphatic *C*-hydroxylation, aromatic *C*-hydroxylation, *N*-dealkylation and *O*-dealkylation, were involved. For validation, the overall accuracies of all four biotransformations exceeded 0.95. For receiver operating characteristic (ROC) analysis, each of these models gave a significant area under curve (AUC) value >0.98. In addition, an external test was performed based on dataset published previously. As a result, 87.7% of the potential SOMs were correctly identified by our four models. In summary, four MMRS-based models were established, which can be used to predict the metabolism mediated by CYP3A4, 2D6, and 2C9 with high accuracy.

## 1. Introduction

Cytochromes P450 enzymes are a family of ubiquitous heme–thiolate proteins that are involved in the metabolism of a large number of Food and Drug Administration (FDA)-approved drugs [1]. As three of the most important isoforms within this family, CYP450 3A4, 2D6, and 2C9 catalyze nearly 50%, 25%, 20% of the therapeutic drugs currently in clinical use, respectively [2,3,4]. Generally, once ingested drugs undergo biotransformation, their therapeutic efficacy always changes [5,6]. Therefore, metabolism mediated by these three isoforms deserve more attention during drug discovery and development.

Commonly, metabolites are determined experimentally. However, for a new chemical entity, it is common that reference compounds for its metabolites are unavailable at the time of analysis. Hence, the usage of in silico procedures to predict the metabolites can be an effective method [7]. To predict the metabolites by in silico methods, defining the sites of metabolism (SOMs) of drugs is usually a starting point. In the current study, the chemical bond within a molecule where the metabolic reaction occurred was defined as the SOM. Therefore, identification of the SOM can provide information about which chemical bond is more susceptible to undergo biotransformation in the body. Often, this information will assist in the process of drug design and optimization. For instance, metabolites of some drugs are likely to diminish or totally lose their therapeutic efficacy; reasonable modification strategies, in ways that retain potency, can be adopted based on information about SOMs [8,9].

During the past decades, there have been continuous attempts in the prediction of metabolites, including ligand-based methods and structure-based methods [10,11,12]. Ligand-based methods rely on the assumption that the metabolic fate of a compound is closely related to its chemical structure and characteristics [13]. In ligand-based methods, structural information of known active and inactive compounds was used to develop prediction models. Now, there are various ligand-based models available to explore potential SOMs, including rule-based methods, pharmacophore-based methods, quantitative structure activity relationship methods, and reactivity-based *ab initio* calculations [4,12]. Models established by ligand-based methods belong to data-driven models, because current advances in metabolism are required to generate prediction models through machine learning [13,14]. For this method, to certain extent, whether the model will be developed successfully directly depends on the quality of original data. However, the existing experimental data is always incomplete, because “false negatives” are unavoidable. Two reasons may account for this. On one side, some SOMs may have not been discovered based on the existing detection technology. On the other side, some SOMs have been discovered but not included in the original data [15,16]. After all, no databases have collected all existing metabolism data. Often, ligand-based methods are simpler and faster than structure-based methods [7]. However, they always fail to consider the interactions between substrates and metabolic enzymes that play a pivotal role in the process of metabolism [17]. Instead, structure-based methods simulate the interactions between substrates and metabolic enzymes successfully through molecular docking and molecular dynamics simulation [18,19]. Both the substrate reactivity and the shape of active sites are taken into consideration. For this method, experimental data is not a prerequisite to create the model, so there are no so-called “false negatives”. Nevertheless, there are indeed some huge challenges for SOM prediction by this method. Structure-based methods rely on the availability of 3D structures of metabolic enzymes to certain extent [20]. However, the 3D structures of many enzymes remain unknown. Meanwhile, protein flexibility and active-site water molecules also bring about great challenges of the application of this method [21,22]. To accurately predict the metabolites, an integrated in silico approach that combines signal from both ligand- and structure-based methods has also been proposed [23,24,25]. However, this hybrid method cannot provide a valid strategy for the problems that both ligand- and structure-based approaches suffer from.

Generally, isoform-unspecific models developed on the basis of ligand-based methods always offer information about the metabolites but fail to give information about the specific isozyme that catalyzes the metabolism [26]. In contrast, isoform-specific models developed by ligand-based methods and models developed by structure-based methods usually only provide information about which site in a molecule is more liable to metabolism by specific CYP isozyme, but cannot determine the metabolic type of SOMs [17,27,28,29,30]. In the current state of the art, a prediction model which can output both signal of biotransformation type of an SOM and specific isozyme catalyzing the corresponding biotransformation always draws more attention. Toward this goal, establishing prediction models based on a microsomal metabolic reaction system (MMRS) may be an effective solution. The nature of biotransformation is the break and formation of a chemical bond in the presence of metabolic enzyme. Therefore, we defined the system formed by the integration of chemical bond data and the corresponding metabolic enzyme information as MMRS. Every chemical bond can be regarded as an MMRS in combination with the corresponding enzyme. The introduction of MMRS will enable us to take the relationship between substrates and metabolic enzymes into account, independent of crystal-derived models. However, another challenge to develop a metabolism prediction model based on existing experimental data is the existence of “false negatives”. To address this issue, a negatives-screening strategy was proposed. By doing so, we attempt to minimize the risks brought by incomplete data. Actually, the complicated in vivo environment also contributes to the metabolism of drugs. Normally, the pH value in vivo ranges from 7.35 to 7.45 [31]. To simulate the in vivo environment as much as possible, all parameters associated with substrate and enzyme were calculated under the condition of pH = 7.4.

This paper presents an attempt to predict metabolism mediated by CYP3A4, 2D6, and 2C9 based on MMRS. Prediction models for the four most significant metabolic reactions were established. Firstly, all potential MMRSs were marked, and these MMRSs were categorized according to the corresponding reaction types. For each reaction type, an individual MMRS dataset was prepared. Each potential MMRS was marked as positive or unlabeled MMRS by comparing the structures of reactants and products. Then, negative MMRSs were acquired through a negatives-screening strategy. Physicochemical properties and topological properties were calculated, under the condition of pH = 7.4, to characterize the reactivity of each MMRS. These properties were used as input to train classifiers, in combination with the enzyme that catalyzes the corresponding biotransformation. To attain the optimal models, a variety of feature selection (FS) algorithms (ChiSquared (CHI), InfoGain (IG), GainRatio (GR), Relief) were used, in combination with multiple classification procedures (Kstar, Bayes (BN), K-nearest neighbours (IBK), C4.5 decision tree (J48), RandomForest (RF), Support vector machines (SVM), AdaBoostM1, Bagging). Ultimately, an optimal model for each biotransformation was attained through a comparison among the combinations of FS algorithms and classification procedures. For validation, the proposed methods were tested on their ability to recognize the reactivity of MMRSs through an independent test set. In addition, an external test was also performed based on dataset published previously. In a word, the current study aims to develop an effective approach to construction of metabolism prediction models, using only the 2D structures of the potential substrates.

## 2. Results

### 2.1. Optimal Model Selection

According to the optimal models screening strategy mentioned in the experimental section, four series of models were achieved for each biotransformation. Then, optimal models were acquired through a comparison among these models.

**Figure 1:**
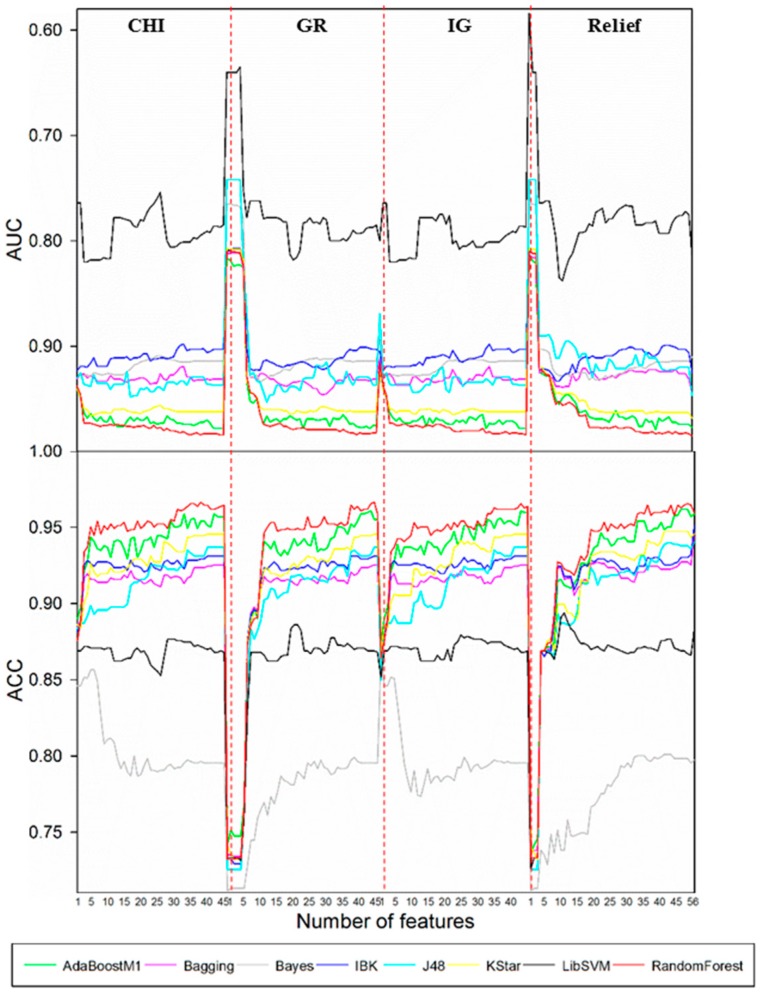
Four series of models for *N*-dealkylation. Here, CHI, GR, IG, and Relief represent models established based on CHI, GR, IG, and Relief FS techniques, respectively. Plots to compare different combinations of FS algorithms and classification algorithms, where both the accuracy (ACC) and area under curve (AUC) derived from 10-fold cross-validation are plotted as functions of the number of selected features. Different classification methods were characterized with different colors.

Such a selection procedure for the case of *N*-dealkylation is demonstrated in Figure 1. Results showed that the best performance was attained by the combination of CHI and RandomForest when the feature number was reduced to 39. Optimal models for other biotransformations were also obtained by the same procedure. Detailed descriptions of these models are demonstrated in Table 1. It turned out that the optimal combinations of FS algorithms and classification procedures varied with the type of biotransformation. Both aliphatic *C*-hydroxylation and *O*-dealkylation attained the best performance by AdaBoostM1 classifier. However, for aromatic *C*-hydroxylation and *N*-dealkylation, RandomForest was more appropriate. For aliphatic *C*-hydroxylation and *N*-dealkylation, the most suitable FS techniques were GR and CHI, respectively. The results were in line with our assumption that there is no classification procedure or FS method that outperformed others in all applications. The optimal FS algorithms for aromatic *C*-hydroxylation and *O*-dealkylation were of particular interest, because they acquired the best performance when no FS was performed. Such a result indicated that in order to accurately predict the metabolites for these two biotransformations, more information may be needed than for aliphatic *C*-hydroxylation and *N*-dealkylation. We surmised that this circumstance may be attributed to the difference among mechanisms of these metabolic reactions. Generally, *N*-dealkylation is easy to predict because of its relatively simple mechanism, but the mechanism of aromatic *C*-hydroxylation is always so complicated that it is difficult to predict the metabolites. There are two alternative mechanisms for *N*-dealkylation: hydrogen atom transfer and single-electron transfer. However, aromatic *C*-hydroxylation proceeds via an epoxide intermediate, and the formation of the epoxide intermediate sometimes undergoes an NIH shift. In addition, direct hydroxylation through an electrophilic aromatic substitution mechanism can also occur. Such a complicated mechanism leads to a low specific SOM pattern [32,33,34,35]. The optimal feature subsets are also included in Table 1. It was not hard to discover that the feature subset of each biotransformation covered both physicochemical properties and topological properties. Therefore, it is safe to speculate that not only physicochemical properties, but also topological properties, are associated with the reactivity of MMRS.

Through a further statistical analysis of the feature subsets, we surprisingly found that 29 out of 56 properties were observed in all cases. Such a result demonstrated that these 29 properties may play a pivotal role in the process of establishing metabolism prediction models. These properties can be further categorized as the following three categories: charge-related (1–10, 13, 17, 18, 21, 22, 25–29), adjacent atom type-related (30–33), and atom hybrid-related (45, 46, 51, 54, 55), which indicated that the reactivity of substrates was attributed to a variety of factors. In addition, we can also make the conclusion that charge-related properties make more of a contribution to the construction of metabolism prediction models than other properties. Twenty out of 29 properties mentioned above were charge-related properties, indicating the importance of charge-related properties.

### 2.2. Estimation of Prediction Models

To assess the overall performance of the optimal models attained above, various indices were calculated. However, it may lead to a misleading evaluation as a result of overfitting that the reliability of one model was evaluated solely by indices related to the training set. Therefore, the test set of each biotransformation was used. In the test set, the reactivity of each MMRS is currently known. They were used as input to validate the models established. Consequently, every MMRS was classified as positive or negative by our models. We believe such a test result will provide us with a more global and unbiased measure of the effectiveness of our models. Both indices associated with training set and test set were shown in Table 2. The accuracy (ACC) of the training sets ranged from 0.928 to 0.984. The sensitivity (SE) and specificity (SP) were generally in excess of 0.953 and 0.921, respectively. For validation, the ACC of each model exceeded 0.95 while the SE varied from 0.958 to 1, and the SP of every model was >0.939. In addition, balanced accuracy (BACC) of the training set and the test set were higher than 0.937 and 0.962, respectively. All these results revealed the reliability and effectiveness of our methods of developing metabolism prediction models.

Receiver operating characteristic (ROC) analysis, another effective approach to the evaluation of the performance of a discriminative classification model, has become increasingly prevalent during the past decades. In this work, ROC analysis for both the training set and the test set was carried out. As a result, we found that every model has a significant area under curve (AUC) value, greater than 0.95, which indicated that our models have a higher reliability. The AUC of each model is also shown in Table 2.

Note that aromatic *C*-hydroxylation, such a complicated biotransformation, was often hard to predict. Almost every prediction model reported has not obtained a satisfactory result. However, our model gave an ACC value of 0.928 with the AUC value = 0.950, and the independent test results were more satisfactory. Therefore, we can speculate that our methods show a superior efficiency for data fitting.

### 2.3. Comparison to Other Models

**Figure 2:**
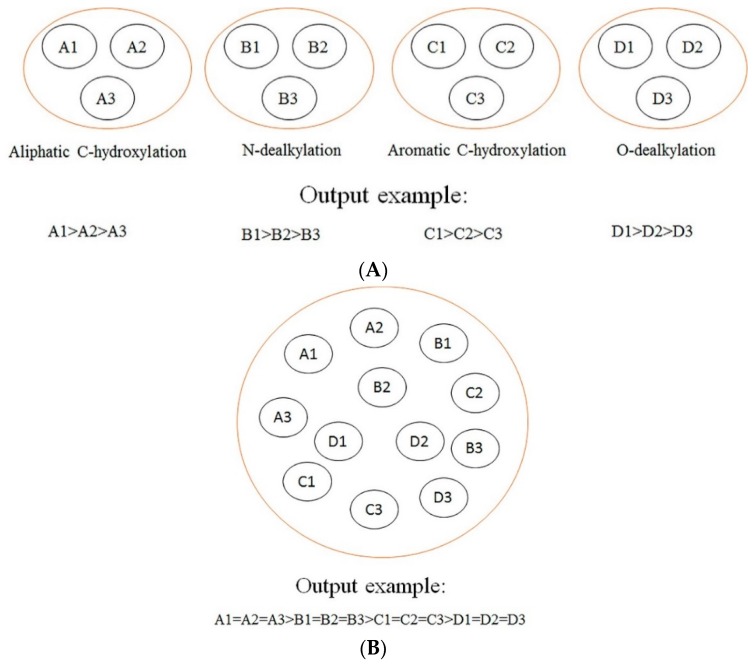

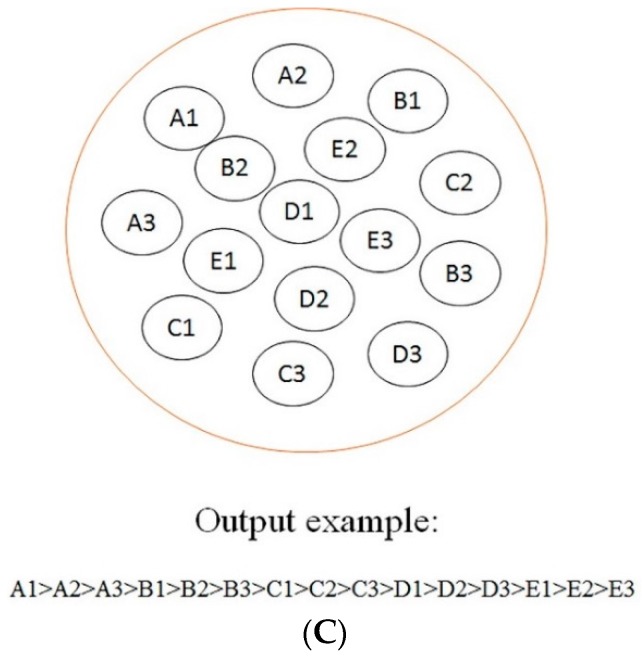
(**A**) Our models; (**B**) rule-based models; (**C**) other ligand-based models and structure-based models. A comparison among our models, rule-based models, ligand-based models, and structure-based models. A1, A2, and A3 represent the sites of metabolism (SOMs) of aliphatic *C*-hydroxylation; B1, B2, and B3 represent the SOMs of *N*-dealkylation; C1, C2, and C3 represent the SOMs of aromatic *C*-hydroxylation; D1, D2, and D3 represent the SOMs of *O*-dealkylation; E1, E2, and E3 represent the SOMs of other type of biotransformations.

In this section, a comparison between our models and other models is conducted. The difference among our models, rule-based models, other ligand-based models (ligand-based models without rules-based models), and structure-based models is demonstrated in Figure 2. As demonstrated in Figure 2, we can find that our models focus on making a comparison among the SOMs of the same type of biotransformation, while the rule-based models tend to make a comparison among the SOMs of different biotransformations, and other ligand-based models and structure-based models aim at ranking all SOMs within a molecule according to their probabilities of undergoing biotransformations. Generally, the consequences of rules-based models, other ligand-based models, and structure-based models are reported in “molecule-scale” (where the top two ranked SOMs of a molecule containing a real SOM was thought to be a correct prediction). However, considering our models were developed from the perspective of reaction type, our model can only provide information about which SOM in a molecule is more liable to undergo certain biotransformation, but cannot provide a top-two metric. In addition, we only considered four biotransformations in the current study. However, other ligand-based models and structure-based models always failed to consider the reaction type when they predicted all possible SOMs. Therefore, there is a difference between our models and these models in terms of the reaction type. It should be noted that the model by Zheng et al. [26]—a model which was designed to predict the SOM of six biotransformations mediated by CYP3A4, 2D6, and 2C9—was also developed from the perspective of reaction type, as we have done. However, it can only provide information about metabolites but cannot point out which isozyme catalyzes the corresponding metabolic reaction. The models developed in the current study give information about the metabolites under the catalysis of specific isozyme. Apparently, there is indeed a challenge to compare the results of Zheng et al. with ours [26]. In summary, considering the difference between results provided by these models and ours, it is difficult to conduct a comparison between our models and others.

Why did we develop a prediction model for each biotransformation but not develop a prediction model for all four biotransformations? Firstly, during the course of the experiment, we tried to develop a prediction model for all four biotransformations, but we discovered that the result was better when we developed a prediction model for each biotransformation. In addition, such a modeling method contributes to reduction of the noise brought on other type of biotransformations. Secondly, it is easy to obtain an optimal model for each biotransformation by developing a prediction model for each biotransformation. From the results of optimal models selection, we can find that the optimal model for each biotransformation was obtained by different combinations of classification procedures and feature selection techniques, and the optimal feature subset also varied with the type of biotransformation. Apparently, such an experimental design not only improves model performance, but also contributes to obtaining the optimal model for each biotransformation.

Also, it should be emphasized that the originality of the current study does not lie in the improvement of other methods. Our research is essentially different from those researches which focus on predicting the SOMs. The main originality of our model lies in providing information about the metabolites and the enzyme which catalyzes the corresponding metabolism simultaneously. It should be noted that for CYP450 3A4, 2D6, and 2C9, currently there are no models reported that can give such detailed metabolism information.

### 2.4. Analysis of the Prediction Model

The originality of the methods proposed in this work was the introduction of MMRS. MMRS can represent the interaction between substrate and enzyme, to certain extent, from the integration of SOM data and enzyme information. It should be emphasized that the ligand-based methods always can only predict where a molecule may be oxidized assuming it is a substrate of certain metabolic enzyme [36]. The proposed of MMRS made it possible to determine the metabolism of drugs and give information about metabolic enzyme simultaneously.

We concur the SMARTCyp authors’ view that false negatives (which are inevitable in the experimental data) always lead to minor signal-to-noise [16]. We believe this is not a secret in the domain of metabolism prediction. However, almost all data-driven models lost sight of this fact. There is no doubt that the higher the quality of original data, the more reliable the models. Therefore, one advantage of the proposed methods was that a negatives-screening strategy was used to reduce the number of false negatives. By doing so, we attempt to increase the signal-to-noise ratio of original data. Another advantage of the methods was that eight classification procedures were implemented to recognize characteristic patterns in combination with four FS methods. As a result, 32 combinations of FS methods and classification procedures were generated. As we all know, there is no classifier procedure or FS method that outperforms others in all domains. Apparently, our methods are more likely to attain the optimal models than those only using one classification or FS method. The results demonstrated in Table 1 are in line with our conjecture.

External validation is always regarded as an effective method to measure the generalization of prediction models. Therefore, the dataset collected by Sheridan et al. was used to validate these models. We believe such an external test can provide us with insight into how these models will perform in realistic problems.

**Figure 3:**
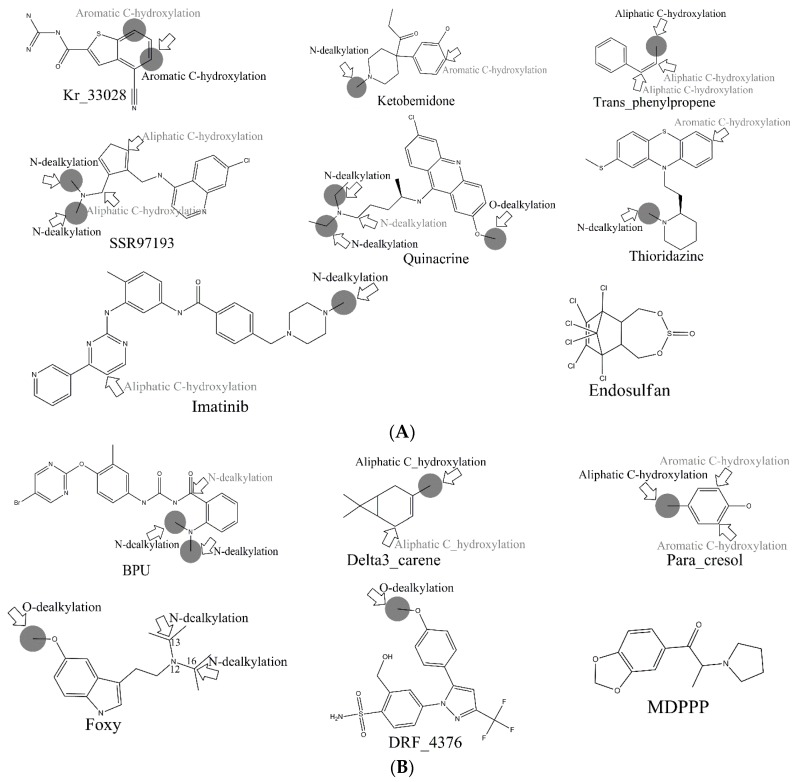

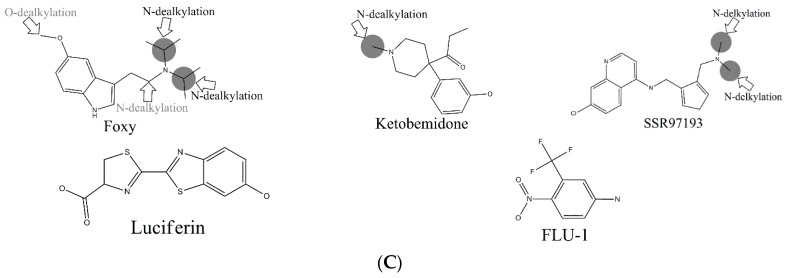
(**A**) CYP450 3A4; (**B**) CYP450 2D6; (**C**) CYP450 2C9. Example molecules in the external set that were well-predicted and poorly-predicted by full models established in this work. Experimentally observed SOMs are indicated with gray solid circles. Predicted SOMs with the microsomal metabolic reaction system (MMRS)-based prediction models are marked with hollow arrows where the designated biotransformations were also recorded. Noted are biotransformations marked with light gray—it is suggested that these SOMs were incorrectly identified. In contrast, black represents the SOMs correctly identified.

Consequently, combing our four models, 760 out of 867 MMRSs were correctly identified with an accuracy value of 0.877 for the external test set. Specifically, all the positive MMRSs were correctly identified for *N*-dealkylation and *O*-dealkylation. Apparently, our models gave a satisfactory performance. However, in common with all models reported, the performance of aromatic *C*-hydroxylation was not so optimistic. Although we have attained the optimal model, the overall accuracy of the external set for aromatic *C*-hydroxylation did not exceed 0.77. Some well-predicted and poorly-predicted compounds are shown in Figure 3. It should be noted that Zheng et al. gave an overall accuracy value of 0.79 for the same test set based on prediction models for six metabolic reactions [26], and neither Sheridan et al. model nor MetaSite can explain more than about 70% of the regioselectivity data [17]. However, because of the difference of biotransformations involved between these models and ours, our models were not able to compare with them.

In the external test set, for the case of endosulfan, MDPPP, luciferin, and FLU-1, there are no positive SOMs within these four compounds. Fortunately, no false positives were introduced in the process of prediction. Similarly, for DRF_4376, ketobemidone, and SSR97193, our prediction results were also consistent with reports in the literature. The metabolism of Foxy, mediated by CYP450 2D6, may be more interesting. According to the records in Sheridan et al., C13-N12-CYP450 2D6 and C16-N12-CYP450 2D6 were two negative MMRSs. However, in our prediction results, they were classified as positive MMRSs. Note that this is not a fault in our models. By consulting original literature, these were confirmed as positive MMRSs which were observed experimentally [14]. However, for other cases, the results were not so accurate: most misclassified MMRSs were attributed to aromatic *C*-hydroxylation. We can be aware of this from the disappointing performance of aromatic *C*-hydroxylation in Figure 3.

Comparing the results of the external test with the independent test, we easily found that there was a difference between the performances for the model of aromatic *C*-hydroxylation. For the independent test, the model’s performance was fairly satisfactory. However, for the external test, the results were not so optimistic. This may be a signal that the generalization ability of this model was not so idealistic. Considering the complicated mechanism of aromatic *C*-hydroxylation, we believe a solution to improve the performance of this model is to consider more properties. After all, the properties considered in this work may be insufficient to completely represent such a complicated mechanism. Certainly, we also believe a more high-quality dataset also contributes to the work.

Another question that should be answered was whether the MMRS can represent the interaction between substrate and metabolic enzyme. As demonstrated in Figure 3, it was not hard to find that the prediction results for ketobemidone, under the catalysis of CYP 3A4 and CYP 2C9, were different. Under the catalysis of CYP 3A4, an *N*-dealkylation metabolite and an aromatic *C*-hydroxylation metabolite were outputted. However, under the catalysis of CYP 2C9, only an *N*-dealkylation metabolite was given. For SSR97193 and Foxy, we also found that the metabolites varied with metabolic enzyme. Therefore, we can speculate that MMRS can represent the interaction between substrate and enzyme to certain extent.

Metabolism prediction models for other reaction types mediated by these enzymes or other enzymes will make a significant breakthrough when sufficient data is available. Furthermore, metabolic pathway predictive model of xenobiotics will also be established based on these metabolism prediction models. However, the development of a web service for metabolism estimating and visualizing may be the first work to carry out.

## 3. Materials and Methods

### 3.1. Dataset and Data Preprocessing

Structures and SOMs of substrates of CYP3A4, 2D6, and 2C9 originated from the work of Sheridan et al [16,17]. All substrates were screened according to the type of atom. Only C, H, O, N, S, P, F, Cl, Br, and I were permitted. In the dataset collected, a substrate of CYP2D6 and 2C9, bortezemib was found to contain boron atom, thus, it was removed. Eventually, we had assembled 394, 123, and 93 substrates for CYP3A4, 2D6, and 2C9, respectively. Then, information about biotransformation type of each SOM was determined one by one through curation of citations provided by Sheridan et al. Descriptive statistics of the metabolic reactions of these substrates are shown in Table 3. The reactions of which count of positive SOM <60 were not considered because these datasets were too small to develop effective models. Finally, four biotransformations with sufficient data were considered in the current study. Visual graphic definitions of these four biotransformations were shown in Table 4. The structures of all substrates are available as an SDF file in supplementary File 1.

For further model validation, the external test set complied by Sheridan et al. was collected [17]. All substrates were also screened according to the type of atom. As mentioned above, substrates only consisting of C, H, O, N, S, P, F, Cl, Br, and I were permitted. The final set consisted of 19 molecules for CYP3A4, 10 molecules for 2D6, and 9 molecules for 2C9. This external test set is also available as an SDF file in supplementary File 1.

### 3.2. Microsomal Metabolic Reaction System

As mentioned in the introduction, every chemical bond can be regarded as an MMRS in combination with the corresponding enzyme that catalyzes the substrate. The pattern of MMRS varies with the reaction type (Table 5). For each MMRS pattern, an individual dataset was prepared. Each MMRS was marked as positive or unlabeled, which depends upon whether the MMRS undergoes biotransformation. According to the available literature reports, if an SOM was experimentally determined as a positive SOM, we marked the corresponding MMRS as a positive MMRS. In contrast, if an SOM has not been experimentally detected as a positive SOM, the corresponding MMRS was marked as an unlabeled MMRS. To achieve negatives, the positive MMRSs and unlabeled MMRSs in each dataset were used as input to train classifier respectively. Eight classification algorithms (Kstar [37], BN [38], IBK [39], J48 [40], RF [41], SVM [42], AdaBoost [43], Bagging [44]) were adopted. These algorithms have been shown effective in various domains of bioinformatics and medicinal chemistry. As a result, eight discriminative models for each reaction type were established by using 10 cross-validations. For each unlabeled MMRS in each dataset, 1 score was recorded when it was predicted as unlabeled by one discriminative model, otherwise a 0 score was recorded. Then, the total score of each unlabeled MMRS was calculated. The unlabeled MMRSs in each dataset for which the score was >5 were treated as negatives. Then, in each dataset, only positive MMRSs and negative MMRSs were retained. In the end, each dataset was randomly divided into training and test sets in the ratio of 4:1. Descriptive statistics of these datasets are demonstrated in Table 5, and all of these MMRSs are detailed in supplementary File 2.

### 3.3. Descriptors of MMRS

Generally, to perform in silico prediction, computational descriptions of entities are necessary. For MMRS, the computational descriptions were implemented by calculating descriptors. The descriptors of MMRS consisted of two parts: descriptors of SOM and descriptors of enzyme. Descriptors of SOM covered both physicochemical property descriptors and topological property descriptors of the corresponding chemical bond. Topological properties always capture information about local structure; by contrast, physicochemical properties are more inclined to characterize the local electronic reactivity. The metabolic enzymes are represented by their names. Altogether, 56 descriptors, including 29 physicochemical property descriptors and 27 topological property descriptors, which have been used to predict the classification of photochemical reactions, were considered in this work [45]. All of these 56 properties are detailed in Table 6. In summary, these properties offered information about charge environment, hybridization environment, atoms’ environment, and bond order. All of the information is closely associated with many features of a molecule, such as ionization constant, electron affinity, and pharmacophore pattern. Many of these features have been confirmed to play an essential role in the process of SOM identification.

The calculation of relevant properties was carried out by the quantum chemistry program Marvin (http://www.chemaxon.com).

### 3.4. Construction of Prediction Models

Machine learning systems are systems that learn from known data and attempt to recognize characteristic patterns. Generally, a model that can be utilized to map the unknown sample into a category was returned after a “learning phase”. For the identification of SOM characteristic patterns, classification is always considered to be an efficient and effective method. However, as many pattern-recognition techniques were originally not designed to cope with large amounts of irrelevant features, the motivation of FS has shifted from being an illustrative example to becoming a real prerequisite for model building. FS was always initiated to avoid overfitting, improve model performance, and search for an optimal feature subset. Therefore, combining classification and FS has become a necessity in many applications.

**Figure 4:**
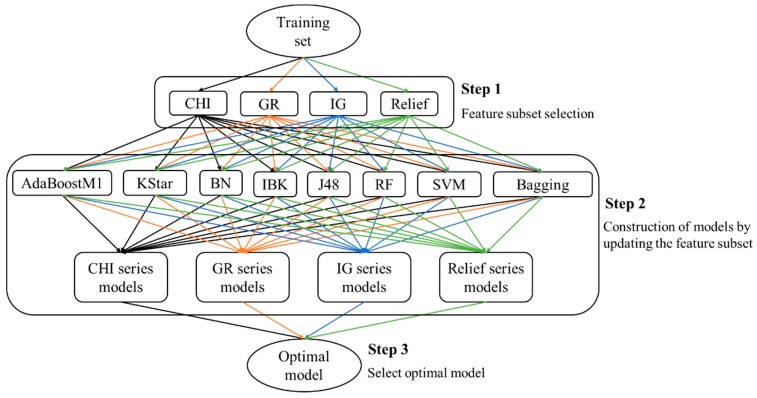
The workflow of optimal model selection.

Currently, so many classifications and FS methods have been proposed. We can claim that each method has its own strength and weakness; no one outperformed others in all applications. Therefore, nobody can tell us which combination of classification and FS is the optimal combination for model building. To attain an optimal model for each biotransformation, eight classifications (AdaBoost, Bagging, Bayes, IBK, J48, KStar, LibSVM, RF) and four FS techniques (CHI [46], GR [47], IG [48], Relief [49]) were adopted (the descriptions of these methods were detailed in Table 7). All of these methods have been proved effective in many fields. As a result, 32 combinations were formed where the optimal combination was attained through a comparison among these combinations. The general procedures of optimal model selection are shown in Figure 4.

Step 1. The goodness of each feature was assessed by our four FS techniques, respectively. All of these four FS techniques are focused on ranking the features in descending order according to their significance scores. The higher the score, the more significant the feature for the training categorization. Features scoring 0 were removed. Other features were retained as feature subsets. It should be noted that all the FS algorithms took place within 10 cross-validations.

Step 2. Feature subsets acquired in step 1 were used as input to train our eight classifiers. Then, the current feature subset was updated by eliminating the feature with the lowest score. The remaining features were used as input to train these eight classifiers. This step was repeated until only one feature was retained. Finally, all the models attained in this step were divided into four categories according to the FS methods. As a result, four series of models for each biotransformation were achieved.

Step 3. In this step, the optimal model for each biotransformation was attained through a comparison among those models attained in step 2. The model which gave the highest ACC and gave an AUC value above 0.9 was selected as the optimal model. All the performances of classifiers were derived from 10 cross-validations.

All the FS methods and classification programs mentioned above were implemented in Waikato Environment for Knowledge Analysis (WEKA) data-mining environment [50]. All the parameters and configurations of the programs were set to default values provided by the WEKA data-mining environment.

### 3.5. Prediction Quality Measurement

In general, accuracy (*ACC*) is a common evaluation indicator of the goodness of a classifier [51]. However, it only offers us rough information about the classifier’s performance. In order to make a comprehensive investigation into the predictive power of the models, we also calculated sensitivity (*SE*), specificity (*SP*), and balanced accuracy (*BACC*). These indices can be expressed in the following form:

(1) $$ACC=\frac{\left(TP+TN\right)}{\left(TP+TN+FP+FN\right)}$$

(2) $$SP=\frac{TN}{\left(TN+FP\right)}$$

(3) $$SE=\frac{TP}{\left(TP+FN\right)}$$

(4) $$BACC=\frac{\left(SE+SP\right)}{2}$$

where *TP*, *TN*, *FP*, *FN* represent the number of true positives, true negatives, false positives and false negatives, respectively.

ROC analysis, which gives further information about the performance of the classification task, was also carried out in this work. In this section, the AUC was calculated as a measure of the performance of the models. The AUC ranges from 0.5 (random classifier, no predictive value) to 1.0 (ideal classifier, perfect prediction). The higher the AUC value, the more reliable the model. Generally, we believe a model has a good predictive power when the corresponding AUC value is >0.9 [52,53].

## 4. Conclusions

In the current study, four MMRS-based metabolism prediction models were established to predict the metabolism mediated by CYP450 3A4, 2D6, and 2C9 for the first time. Four major biotransformations, including aliphatic *C*-hydroxylation, aromatic *C*-hydroxylation, *N*-dealkylation, and *O*-dealkylation, were involved. A set of physicochemical properties and topological properties were calculated, under the condition of pH = 7.4, for each SOM to represent the reactivity of a potential MMRS. In addition, a negatives-screening strategy was introduced to increase the signal-to-noise ratio. By incorporating the use of multiple FS techniques and multiple classification procedures, the optimal model for each metabolic reaction was attained. Both the internal and external validation results indicated that the models were considerable successes. We believe such a novel method can provide a wider insight into metabolism information in the process of drug discovery and development, including metabolites and the enzyme involved in the metabolism process of drugs. Furthermore, our work will also lay a foundation for the construction of metabolic pathways predictive model.

## Figures and Tables

**Table 1:** Optimal models selection results.

Reaction Type	Classifier	FS	No. of Features	Scheme
I	AdaBoostM1	GR	31	1–10,13,17,18,21,22,25–33,35,36,45,46,51,54,55
II	RandomForest	none	56	1–56
III	RandomForest	CHI	39	1–33,38,45,46,51,54,55,
IV	AdaBoostM1	none	56	1–56

ID in scheme corresponds to feature ID in Table 6.

**Table 2:** Prediction quality of the four optimal models, in terms of different statistic indices.

Data Set	Reaction Type	SE	SP	ACC	BACC	AUC
Training set	I	0.956	0.983	0.976	0.970	0.984
	II	0.953	0.921	0.928	0.937	0.950
	III	0.972	0.965	0.967	0.969	0.984
	IV	0.978	0.987	0.984	0.983	0.993
Test set	I	0.958	0.989	0.981	0.974	0.995
	II	1.000	1.000	1.000	1.000	1.000
	III	0.985	0.939	0.950	0.962	0.984
	IV	1.000	1.000	1.000	1.000	1.000

SE, SP, ACC, BACC, and AUC represent the sensitivity, specificity, accuracy, balanced accuracy and area under curve, respectively.

**Table 3:** Description of the datasets collected from the literature.

Reaction Type	No. of Positive SOM	Percentage (%)
Aliphatic *C*-hydroxylation	1411	61
Aromatic *C*-hydroxylation	314	13.6
*N*-dealkylation	347	15
*O*-dealkylation	137	5.9
*S*-oxidations	57	2.5
*N*-oxidations	27	1.2
Desulfurization	7	0.3
Others	15	0.6

**Table 4:** Visual graphic definitions of four biotransformations mediated by CYP450 3A4, 2D6, and 2C9.

ID	Biotransformation	Definition
I	Aliphatic *C*-hydroxylation	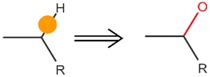
II	Aromatic *C*-hydroxylation	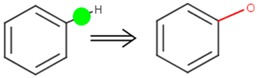
III	*N*-Dealkylation	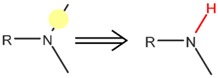
IV	*O*-Dealkylation	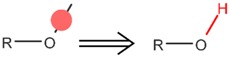

Potential SOM of each biotransformation is marked with solid circle by different colors.

**Table 5:** Descriptive statistics of MMRS datasets used for modeling.

Reaction Type	MMRS Pattern	NO. of MMRS		
		Unlabeled	Negtive	Postive
I	Aliphatic C–H-enzyme	4233	4091	1411
II	Aromatic C–H-enzyme	942	828	314
III	C–N-enzyme	1041	961	347
IV	C–O-enzyme	411	374	137

Unlabeled, Negative, and Positive represent unlabeled MMRS, negative MMRS, and positive MMRS, respectively.

**Table 6:** Descriptor definitions.

ID	Lable	Description
Physicochemical descriptors		
1	qtot_A	total charge of A
2	qsigma_A	σ charge of A
3	qpi_A	π charge of A
4	pol_A	polarizability of A
5	oensigma_A	σ orbital-electronegativity of A
6	oenpiA	π orbital-electronegativity of A
7	pichgdens_A	π charge density of A
8	totchgdens_A	total charge density of A
9	qtot_B	total charge of B
10	qsigma_B	σ charge of B
11	qpi_B	π charge of B
12	pol_B	polarizability of B
13	oensigma_B	σ orbital-electronegativity of B
14	oenpi_B	π orbital-electronegativity of B
15	pichgdens_B	π charge density of B
16	totchgdens_B	total charge density of B
17	maxqtot_A, (Except B)	maximum charge of A neighbors
18	minqtot_A, (Except B)	minimum charge of A neighbors
19	maxqtot_B, (Except A)	maximum charge of B neighbors
20	minqtot_B, (Except A)	minimum charge of B neighbors
21	maxpol_A, (Except B)	maximum polarizability of A neighbors
22	minpol_A, (Except B)	minimum polarizability of A neighbors
23	maxpol_B, (Except A)	maximum polarizability of B neighbors
24	minpol_B, (Except A)	minimum polarizability of B neighbors
25	dqtot	difference of total charges
26	dqsigma	difference of σ charges
27	dqpi	difference of π charges
28	doensigma	difference of σ orbital-electronegativity
29	doenpi	difference of π orbital-electronegativity
Topological descriptors		
30	Hn_A, (Except B)	number of H-atoms bonded to A
31	Cn_A, (Except B)	number of C-atoms bonded to A
32	Nn_A, (Except B)	number of N-atoms bonded to A
33	On_A, (Except B)	number of O-atoms bonded to A
34	Pn_A, (Except B)	number of P-atoms bonded to A
35	Sn_A, (Except B)	number of S-atoms bonded to A
36	Xn_A, (Except B)	number of halogen atoms bonded to A
37	Hn_B, (Except A)	number of H-atoms bonded to B
38	Cn_B, (Except A)	number of C-atoms bonded to B
39	Nn_B, (Except A)	number of N-atoms bonded to B
40	On_B, (Except A)	number of O-atoms bonded to B
41	Pn_B, (Except A)	number of P-atoms bonded to B
42	Sn_B, (Except A)	number of S-atoms bonded to B
43	Xn_B, (Except A)	number of halogen atoms bonded to B
44	Csp1_A	A is C sp1
45	Csp2_A	A is C sp2
46	Csp3_A	A is C sp3
47	Csp1_B	B is C sp1
48	Csp2_B	B is C sp2
49	Csp3_B	B is C sp3
50	Csp1_neig A	number of C sp1 neighbors of A
51	Csp2_neig A	number of C sp2 neighbors of A
52	Csp3_neig A	number of C sp3 neighbors of A
53	Csp1_neig B	number of C sp1 neighbors of B
54	Csp2_neig B	number of C sp2 neighbors of B
55	Csp3_neig B	number of C sp3 neighbors of B
56	boord	bond order

A and B are two atoms located at either end of the chemical bond.

**Table 7:** Overview of FS algorithms and classifier algorithms.

Name	Full Name	Description
CHI	ChiSquared	Evaluates the worth of an attribute by computing the value of the chi-squared statistic with respect to the class.
GR	GainRatio	Evaluates the worth of an attribute by measuring the gain ratio with respect to the class.
IG	InfoGain	Evaluates the worth of an attribute by measuring the information gain with respect to the class.
Relief	Relief	Evaluates the worth of an attribute by repeatedly sampling an instance and considering the value of the given attribute for the nearest instance of the same and different class.
AdaBoostM1	AdaBoostM1 with C4.5 as its base-level classifier	The purpose of AdaBoostM1 is to find a highly accurate classification rule by combining many weak classifiers, each of which may be only moderately accurate.
KStar	KStar	An instance-based classifier that is the class of a test instance is based on the class of those training instances similar to it, as determined by some similarity functions.
BN	Bayes	This classifier learns from training data, the conditional probability of each attribute A_i_ given the class label C. Classification is then done by applying Bayes rule to compute the probability of C given the particular instance of A_1_, A_n_, and then predicting the class with the highest posterior probability.
IBK	K-nearest neighbours	The IBK classifier is commonly based on the Euclidean distance between a test sample and the specified training samples. The classification rule is to assign a test sample to the majority category label of its k nearest training samples.
J48	C4.5 decision tree	This classifier is generated based on the instances, and focuses on deducing classification rules represented by decision tree from a group of out-of-order or out-of-rule samples.
RF	RandomForest	Random forest classifiers work by growing a predetermined number of decision trees simultaneously. A test instance is run on all the decision trees grown in the forest. Each tree’s classification of the test instance is recorded as a vote. The votes from all trees are aggregated and the test instance is assigned to the class that receives the maximum vote.
SVM	Support vector machines	Input vectors are non-linearly mapped to a very high-dimension feature space. Where a linear decision surface is constructed. The goal of the SVM algorithm is to find an optimal hyperplane that separates the training samples by a maximal margin, with all positive samples lying on one side and all negative samples lying on the other side based on statistical learning theory. In this work, LibSVM was adopted.
Bagging	Bagging with KNN as its base-level classifier	Bagging predictors is a method for generating multiple versions of a predictor and using these to get an aggregated predictor. The multiple versions are formed by making bootstrap replicates of the learning set and using these as new learning sets.

## Supplementary Materials

Supplementary materials can be found at www.mdpi.com/1422-0067/17/10/1686/s1.

## References

[1] Matlock M.K., Hughes T.B., Swamidass S.J. (2015). XenoSite server: A web-available site of metabolism prediction tool. Bioinformatics.

[2] Mo S.L., Zhou Z.W., Yang L.P., Wei M.Q., Zhou S.F. (2009). New insights into the structural features and functional relevance of human cytochrome P450 2C9. Part I. Curr. Drug Metab..

[3] Zhou S.F., Liu J.P., Lai X.S. (2009). Substrate specificity, inhibitors and regulation of human cytochrome P450 2D6 and implications in drug development. Curr. Med. Chem..

[4] Dai Z.R., Ai C.Z., Ge G.B., He Y.Q., Wu J.J., Wang J.Y., Man H.Z., Jia Y., Yang L. (2015). A mechanism-based model for the prediction of the metabolic sites of steroids mediated by cytochrome P450 3A4. Int. J. Mol. Sci..

[5] Bo W., Li M., Zhu M. (2008). Bioactivation of the tricyclic antidepressant amitriptyline and its metabolite nortriptyline to arene oxide intermediates in human liver microsomes and recombinant P450s. Chem. Biol. Interact..

[6] Dockens R.C., Salazar D.E., Fulmor I.E., Wehling M., Arnold M.E., Croop R. (2006). Pharmacokinetics of a newly identified active metabolite of buspirone after administration of buspirone over its therapeutic dose range. J. Clin. Pharmacol..

[7] Nielsen L.M., Linnet K., Olsen L., Rydberg P. (2014). Prediction of cytochrome P450 mediated metabolism of designer drugs. Curr. Top. Med. Chem..

[8] Liederer B.M., Borchardt R.T. (2006). Enzymes involved in the bioconversion of ester-based prodrugs. J. Pharm. Sci..

[9] Wiltshire D.H., Hirankarn S., Farrell C., Paya C., Pescovitz M.D., Humar A., Dominguez E., Washburn K., Blumberg E., Alexander B. (2005). Pharmacokinetic profile of ganciclovir after its oral administration and from its prodrug, valganciclovir, in solid organ transplant recipients. Clin. Pharm..

[10] Stjernschantz E., Vermeulen N.P., Oostenbrink C. (2008). Computational prediction of drug binding and rationalisation of selectivity towards cytochromes P450. Expert Opin. Drug Metab. Toxicol..

[11] Akos T., Keseru G.R.M. (2011). In silico site of metabolism prediction of cytochrome P450-mediated biotransformations. Expert Opin. Drug Metab. Toxicol..

[12] Kirchmair J., Williamson M.J., Tyzack J.D., Tan L., Bond P.J., Bender A., Glen R.C. (2012). Computational prediction of metabolism: Sites, products, SAR, P450 enzyme dynamics, and mechanisms. J. Chem. Inf. Model..

[13] Melofilho C.C., Braga R.C., Andrade C.H. (2014). Advances in methods for predicting phase I metabolism of polyphenols. Curr. Drug Metab..

[14] Zaretzki J., Rydberg P., Bergeron C., Bennett K.P., Olsen L., Breneman C.M. (2012). RS-predictor models augmented with SMARTCyp reactivities: Robust metabolic regioselectivity predictions for nine CYP isozymes. J. Chem. Inf. Model..

[15] Hennemann M., Friedl A., Lobell M., Keldenich J., Hillisch A., Clark T., Göller A.H. (2009). CypScore: Quantitative prediction of reactivity toward cytochromes P450 based on semiempirical molecular orbital theory. Chemmedchem.

[16] Rydberg P., Gloriam D.E., Zaretzki J., Breneman C., Olsen L. (2010). SMARTCyp: A 2D method for prediction of cytochrome P450-mediated drug metabolism. ACS Med. Chem. Lett..

[17] Sheridan R.P., Korzekwa K.R., Torres R.A., Walker M.J. (2007). Empirical regioselectivity models for human cytochromes P450 3A4, 2D6, and 2C9. J. Med. Chem..

[18] Wang R., Lai L., Wang S. (2002). Further development and validation of empirical scoring functions for structure-based binding affinity prediction. J. Comput. Aided Mol. Des..

[19] Shan Y., Kim E.T., Eastwood M.P., Dror R.O., Seeliger M.A., Shaw D.E. (2011). How does a drug molecule find its target binding site?. J. Am. Chem. Soc..

[20] Crivori P., Poggesi I. (2006). Computational approaches for predicting CYP-related metabolism properties in the screening of new drugs. Eur. J. Med. Chem..

[21] Li J., Cai J., Su H., Du H., Zhang J., Ding S., Liu G., Tang Y., Li W. (2016). Effects of protein flexibility and active site water molecules on the prediction of sites of metabolism for cytochrome P450 2C19 substrates. Mol. Biosyst..

[22] Sheng Y., Chen Y., Lei W., Liu G., Li W., Yun T. (2014). Effects of protein flexibility on the site of metabolism prediction for CYP2A6 substrates. J. Mol. Graph. Model..

[23] De Groot M.J., Ackland M.J., Horne V.A., Alex A.A., Jones B.C. (1999). Novel approach to predicting P450-mediated drug metabolism:  Development of a combined protein and pharmacophore model for CYP2D6. J. Med. Chem..

[24] De Groot M.J., Ackland M.J., Horne V.A., Alex A.A., Jones B.C. (1999). A novel approach to predicting P450-mediated drug metabolism. CYP2D6 catalyzed *N*-dealkylation reactions and qualitative metabolite predictions using a combined protein and pharmacophore model for CYP2D6. J. Med. Chem..

[25] Kingsley L.J., Wilson G.L., Essex M.E., Lill M.A. (2015). Combining structure- and ligand-based approaches to improve site of metabolism prediction in CYP2C9 substrates. Pharm. Res..

[26] Zheng M., Luo X., Shen Q., Wang Y., Du Y., Zhu W., Jiang H. (2009). Site of metabolism prediction for six biotransformations mediated by cytochromes P450. Bioinformatics.

[27] Danielson M.L., Desai P.V., Mohutsky M.A., Wrighton S.A., Lill M.A. (2011). Potentially increasing the metabolic stability of drug candidates via computational site of metabolism prediction by CYP2C9: The utility of incorporating protein flexibility via an ensemble of structures. Eur. J. Med. Chem..

[28] Kumar R. (2015). Prediction of metabolism of drugs using artificial intelligence: How far have we reached?. Curr. Drug Metab..

[29] Rudik A., Dmitriev A., Lagunin A., Filimonov D., Poroikov V. (2015). SOMP: Web server for in silico prediction of sites of metabolism for drug-like compounds. Bioinformatics.

[30] Matlock K.M., Hughes B.T., Swamidass J.S. (2014). XenoSite-Server: A web-available site of metabolism prediction tool. Bioinformatics.

[31] Barfod C., Danker J.K., Sölétormos G., Berlac P.A., Lippert F., Lundstrøm L.H., Antonsen K., Kai H.W.L. (2012). The formation and design of “the Acute Admission Database”—A database including a prospective, observational cohort of 6279 patients triaged in the Emergency Department in a larger Danish hospital. Scand. J. Trauma Resusc. Emerg. Med..

[32] Gasteiger J. (2007). Modeling chemical reactions for drug design. J. Comput. Aided Mol. Des..

[33] Guroff G., Daly J.W., Jerina D.M., Renson J., Witkop B., Udenfriend S. (1967). Hydroxylation-induced migration: The NIH shift. Recent experiments reveal an unexpected and general result of enzymatic hydroxylation of aromatic compounds. Science.

[34] Korolev D., Balakin K.V., Nikolsky Y., Kirillov E., Ivanenkov Y.A., Savchuk N.P., Ivashchenko A.A., Nikolskaya T. (2003). Modeling of human cytochrome P450-mediated drug metabolism using unsupervised machine learning approach. J. Med. Chem..

[35] Li D., Wang Y., Yang C., Han K. (2009). Theoretical study of *N*-dealkylation of *N*-cyclopropyl-*N*-methylaniline catalyzed by cytochrome P450: Insight into the origin of the regioselectivity. Dalton Trans..

[36] Cruciani G., Carosati E., De B.B., Ethirajulu K., Mackie C., Howe T., Vianello R. (2005). MetaSite: Understanding metabolism in human cytochromes from the perspective of the chemist. J. Med. Chem..

[37] Cleary J.G., Trigg L.E., Armand P., Stuart R. (1996). K*: An Instance-Based Learner using an Entropic Distance Measure. Machine Learning Proceedings 1995: Proceedings of the Twelfth International Conference on Machine Learning.

[38] Goldszmidt M. (1997). Bayesian network classifiers. Mach. Learn..

[39] Chavan S., Nicholls I.A., Karlsson B.C., Rosengren A.M., Ballabio D., Consonni V., Todeschini R. (2014). Towards global QSAR model building for acute toxicity: Munro database case study. Int. J. Mol. Sci..

[40] Hu Y.J., Ku T.H., Jan R.H., Wang K., Tseng Y.C., Yang S.F. (2012). Decision tree-based learning to predict patient controlled analgesia consumption and readjustment. BMC Med. Inform. Decis. Mak..

[41] Jayaraj P.B., Ajay M.K., Nufail M., Gopakumar G., Jaleel U.C. (2016). GPURFSCREEN: A GPU based virtual screening tool using random forest classifier. J. Cheminform..

[42] Chang C.C., Lin C.J. (2011). LIBSVM: A library for support vector machines. ACM Trans. Intell. Syst. Technol..

[43] Romero E., Màrquez L.S., Carreras X. (2004). Margin maximization with feed-forward neural networks: A comparative study with SVM and AdaBoost. Neurocomputing.

[44] Breiman L. (1996). Bagging predictors. Mach. Learn..

[45] Zhang Q.Y., Aires de Sousa J. (2005). Structure-based classification of chemical reactions without assignment of reaction centers. J. Chem. Inform. Model..

[46] Hernández-Torruco J., Canul-Reich J., Frausto-Solís J., Méndez-Castillo J.J. (2014). Feature selection for better identification of subtypes of Guillain-Barré syndrome. Comput. Math. Methods Med..

[47] Ramani R.G., Jacob S.G. (2013). Improved classification of lung cancer tumors based on structural and physicochemical properties of proteins using data mining models. PLoS ONE.

[48] Zheng Z., Wu X., Srihari R. (2010). Feature selection for text categorization on imbalanced data. ACM Sigkdd Explor. Newsl..

[49] Pang Z., Zhu D., Chen D., Li L., Shao Y. (2015). A computer-aided diagnosis system for dynamic contrast-enhanced MR images based on level set segmentation and ReliefF feature selection. Comput. Math. Methods Med..

[50] Hall M., Frank E., Holmes G., Pfahringer B., Reutemann P., Witten I.H. (2010). The WEKA data mining software: An update. ACM Sigkdd Explor. Newsl..

[51] Gruss S., Treister R., Werner P., Traue H.C., Crawcour S., Andrade A., Walter S. (2015). Pain intensity recognition rates via biopotential feature patterns with support vector machines. PLoS ONE.

[52] Fawcett T. (2010). ROC graphs: Notes and practical considerations for researchers. Mach. Learn..

[53] Fawcett T. (2006). An introduction to ROC analysis. Pattern Recognit. Lett..

